# Implementation of mhGAP in Mozambique: integrating epilepsy care into the primary health care system

**DOI:** 10.1186/s13033-019-0296-5

**Published:** 2019-05-29

**Authors:** Palmira Fortunato Dos Santos, Vasco Cumbe, Maria Lídia Gouveia, Capucine de Fouchier, Dirk Teuwen, Tarun Dua

**Affiliations:** 1 Mental Health Department, Center for Applied Psychology and Psychometric Tests, Ministry of Health of Mozambique, Rua de Nachingwea Nº 486, Maputo, Mozambique; 2 Provincial Health Directorate of Sofala, Beira Central Hospital, Beira, Mozambique; 3 Mental Health Department, Ministry of Health, Maputo, Mozambique; 4 World Health Organization, Geneva, Switzerland; 5 UCB, Brussels, Belgium

**Keywords:** Epilepsy, mhGAP, Treatment gap, Task-shifting, Task-sharing, Primary health care, Antiepileptic, Mozambique

## Abstract

**Background:**

Epilepsy remains the most frequent diagnosis in Psychiatric and Mental Health Services in Mozambique. Because it is a major concern, in 2013 a Program for “*Reducing the Epilepsy Treatment gap*” was launched in 16 districts of five provinces covering a population of over 1.9 million. Using the WHO Mental Health Gap Program (mhGAP), a pilot Program was developed to provide effective quality care and treatment for people with epilepsy at primary health care level. Implementation was against a background of a shortage of human resources trained to address epilepsy and difficulties in the availability of antiepileptic medicines.

**Methods:**

The first step for implementation was advocacy from the Government level to relevant stakeholders in the community. mhGAP training materials were translated and adapted to the local context. Non-specialists health providers and community health workers were trained and supervised regularly. Population awareness raising and community involvement were key for acceptance of the Program.

**Results:**

After 4 years of implementation, 177 health professionals and 1161 community health workers were trained and ensured services delivery for people living with epilepsy (PwE). The implementation led to 89,869 consultations, representing an increase of 67% since the Program’s inception. From 2015 to 2017 a total of 13,563 new cases were attended and the treatment gap was reduced from 99 to 96%. More than 60% of the new cases are children and adolescents. Awareness actions reached more than 14,000 people per year using all available broadcast means. Preliminary positive results were used as evidence for the Ministry of Health (MoH) to increase the purchase of antiepileptic drugs and improve delivery at district level.

**Discussion:**

mhGAP is an important tool for reducing the treatment gap in low-income countries. Adapting guidelines to the country context and involving community stakeholders are key for Program sustainability. As in other settings, the strategy was cost-effective resulting in an increase in new cases and follow-up consultations.

**Conclusions:**

Implementation of an adapted mhGAP strategy and the involvement of community stakeholders and commitment of the MoH resulted in significant increase in the number of PwE attending outpatient services in primary health care facilities.

## Introduction

### Background

Epilepsy affects more than 65 million people worldwide [1] and represents 0.6% of disability adjusted life years [2]. About 80% of People living with Epilepsy (PwE) live in resource poor countries where the incidence is 2–3 times higher than in developed countries [3–6]. Most PwE do not receive appropriate treatment although there is evidence of cost-effective medicines to control seizures [4, 6, 7].

The treatment of PwE has been neglected in the public health programs in spite of the evidence on impact and the burden of this disease on individuals, families and society. Over 70–80% of the epilepsy population can lead normal lives if diagnosed and treated properly [1, 7]. However, the treatment gap, defined as the number of people with an illness, disease, or disorder who need treatment but do not get it [7–9] is about 80–98% in developing countries [10].

The main reasons for the treatment gap include low priority for epilepsy in the public health agenda [6, 7], inadequate training of health professionals, unavailability of anti-epileptic drugs, lack of community awareness and people’s understanding, beliefs, and superstitions about the etiology of seizures. These factors partly explain why many PwE do not go to a health care facility for treatment and prefer traditional medicine practitioners as a first choice at community level [11–14].

Epilepsy, among other mental and neurological disorders, can be identified and treated in primary health care settings in resource poor countries at low cost [4]. Diagnosis and treatment of epilepsy can be done by trained non-specialized mental health personnel without the need for advanced technology or equipment [5, 7]. Most people with epilepsy (65%) can be treated with affordable antiepileptic drugs [15].

To increase coverage of mental and neurological disorders, including epilepsy care worldwide, in 2008 the World Health Organization (WHO) launched the mental health Gap Action Program (mhGAP) to reduce the burden of mental and neurological disorders including epilepsy. The Program includes development and evaluation of a model of epilepsy care that incorporates various strategies combining skill enhancement of health professionals (experts and non-specialists), participation of people with epilepsy, their families and the community to ensure that every man, woman, and child with epilepsy receive treatment in many poor and developing countries [7, 16].

Mozambique is a low-income country ranking 184th in Human Development Index, located in the southern Africa surrounded by six countries, namely Tanzania, Malawi, Zambia, Zimbabwe, South Africa and Swaziland. The country’s population is 25,727,919 inhabitants with a life expectancy of 51.8 years for women and 47.1 for men. The literacy rate is 49.6% and the official language is Portuguese [17].

Based on the actual population size, it is estimated that over 411,647 people in Mozambique live with epilepsy, considering a prevalence of 1.6% [18]. It is the main cause of consultations in Mental Health Services with records of 15,545 epilepsy consultations conducted throughout the country in 2014. These figures represent 47.6% of the overall mental health consultations in the country that year [19]. In Mozambique as in many low and middle income countries, epilepsy represents a burden and is a priority for the health sector [11, 20].

The National Health Service provides free coverage for the general population with special care for those with chronic diseases. There are 1377 health facilities in the country of which 1249 are primary health care facilities. Only 153 health facilities offer mental health care and treatment and most of these are not in primary health care. For mental health admission there are only 84 beds per 100,000 inhabitants [21]. Prevalence of mental disorders is not well established for the country. Prevalence for psychoses is higher in rural areas than in urban areas (4.4% versus 1.6%), mental retardation (1.9% versus 1.3%) and seizure disorders (4.0% versus 1.6%) according to the only prevalence study published for the country [18]. The 1.6% prevalence for epilepsy is used as reference for this study.

A situation analysis study was conducted to evaluate epilepsy in the five provinces of Mozambique selected for this study. This study concluded that stigma and discrimination were relevant barriers for treatment seeking. There were issues on anti-epileptic drugs availability in all health facilities and there was also a need to estimate the treatment gap. A total of 798 health workers were in place and only 874 patients attended treatment for epilepsy in the previous 12 months [20].

As for human resources, Mozambique has 507 doctors per 100,000 inhabitants. The situation for mental health is critical considering that the core of mental health providers is of 386 professionals (13 psychiatrists, 109 psychologists, 241 psychiatric technicians and 23 occupational therapists) for over 25 million inhabitants [21].

To overcome the human resources problem in the health system, Mozambique has adopted the task-shifting/sharing strategy, training mid-level health professionals (equivalent to 12th grade or technical degree) to take some of the tasks of specialists all over the country. Courses of 2 to 3 years duration offered in the Health Sciences Institutes train technicians of surgery, general medicine, psychiatry, pharmacy, laboratory, preventive medicine, nutrition, anesthesiology, physiotherapy and radiology. These professionals are placed in primary and secondary health care facilities working under the supervision of the few specialists in the country.

The psychiatric technicians are trained during a 2½ years course and play the role of the psychiatrist prescribing psychotropic drugs and referring severe and/or complicated cases to the reference hospitals. They are trained to diagnose and treat all mental disorders and epilepsy and cover the secondary and tertiary health care level facilities such as the district, general, and provincial hospitals [21].

Except for Maputo, the capital of the country, primary health care facilities do not have mental health services due to the scarcity of human resources, including the psychiatric technicians. To mitigate this problem, mhGAP guidelines for epilepsy were implemented to reduce the treatment gap by using primary health care providers (general medicine technicians and nurses) as an extension of the psychiatric technicians [17].

This paper aims to present a summary of the main achievements of the mhGAP Epilepsy Program implemented in Mozambique from 2014 to 2017.

## Methods

Fifty-six health facilities from 16 districts were selected from a pool of 201 units of five provinces—Niassa, Nampula, Zambézia, Sofala and Gaza—covering the Southern, Central and Northern regions of Mozambique. Inclusion criteria for districts were the number of habitants (these are among the most populous districts in the country), the high prevalence of epilepsy cases when compared to other districts and the presence of a psychiatric technician coordinating mental health services in primary care. Niassa Province was included in the pilot Program due to the high rate of new cases reported yearly in a single district, which fulfills all the inclusion criteria (Table 1).

**Table 1:** Districts and health facilities selection

Province	Nr. of districts		Nr. of health facilities	
	Total (province)	Selected	Total (district)	Selected
Nampula	18	4 (22%)	37	13 (35%)
Zambézia	22	4 (18%)	60	13 (22%)
Sofala	13	4 (30%)	56	12 (21%)
Gaza	14	3 (21%)	36	11 (31%)
Niassa	16	1 (6%)	12	7 (58%)
Total	73	16 (22%)	201	56 (28%)

Mozambican neurologists, psychiatrists and psychologists adapted and used the mhGAP tools for training health professionals not specialized in mental health and community health workers. Training included improving psychiatric technicians’ skills for diagnosis, treatment and follow-up of epilepsy patients. Materials translated and adapted included: (i) the mhGAP-IG epilepsy module and other relevant modules; (ii) training material for non-specialist health professionals; (iii) training materials for community health workers; (iv) training manuals for trainers; (v) Mozambique Manual of Psychiatry and Mental Health (updated and adapted from mhGAP guidelines); and, (vi) training manual for supervisors (last adaptation during the training of trainers).

A cascade model of training was undertaken to start the Epilepsy Program. This included training of 14 trainers and supervisors (6 psychiatrists, 2 neurologists, 2 clinical psychologists and 4 psychiatric technicians) and training of 163 primary care providers. 84% of these professionals are general medicine technicians (n = 81), general nurses (n = 28), medical doctors (n = 21), maternal and child health nurses (n = 8) and psychologist (n = 1), reinforcing task-shifting as a strategy to improve health care and services delivery in low resources settings. Psychiatric technicians make up 16% of the health professionals trained (n = 24).

Refreshing training for 70 non-specialists on epilepsy management was provided after 6 months of intervention to reinforce the quality of service delivery when necessary (Table 2). The 6 months interval was defined for convenience to ensure the services are appropriately delivered.

**Table 2:** Number of health workers trained

Trainings per province	New training	Refreshing
Trainers and supervisors		
Maputo	14	0
Primary care providers		
Gaza	32	14
Nampula	46	23
Niassa	19	6
Sofala	29	17
Zambezia	37	10
Total	177	70

A total of 409 support and supervision visits were conducted: 107 from the national team and 369 from the provincial and district focal points. These visits included technical support, review of difficult cases, follow-up of back-referral cases, advocacy on medicines availability and stocks checks and advocacy with community and district leadership. Support visits to Mozambique by WHO advisers were conducted every year for technical support, planning, budgeting and data analysis.

In addition, 1161 community health workers were trained to be engaged in local health teams to track cases in the community, promote mental health with specific information on epilepsy, refer cases to health facilities and support follow-up at community level (Table 3). This number is insufficient considering the size of the population in the catchment area of the health facilities (1,992,816 inhabitants). Community networks and radio broadcast were also used as a way to reach the targeted population.

**Table 3:** Number of CHW trained in epilepsy per type

Type of community health workers	Number
Community health activists	457
Community leaders	46
Faith leaders	151
Prevention and promotion health workers	146
Journalists	5
Preventive health and promotion workers	18
Students	22
Teachers	116
Traditional healers and leaders	127
Other	73
Total	1161

Traditional healers were involved from the beginning capitalizing on the importance of their position within the communities. Of the new cases, 176 were referred by traditional healers since the Program inception.

The Epilepsy Program developed Information, Education, and Communication (IEC) materials to raise community and public awareness on epilepsy, reduce stigma and inform the targeted population about conventional treatments for epilepsy. This included identifying and contracting various community groups, gathering information on attitudes, stigma and associated factors related to epilepsy, developing educational materials, and developing and executing awareness and educational campaigns.

An initial series of public education and promotional materials, including posters, brochures and videos were produced and translated into local languages for wider dissemination. Community radio, television and other community-based interventions were also conducted involving meetings with community members.

National radio and television channels with national coverage, an interactive group in social networks and a national awareness campaign were used to spread messages and for dissemination of the Epilepsy Program.

All planning and coordination of the pilot Program was carried out by the Mental Health Department at the Ministry of Health and the Provincial Health Directorates. Each of the selected provinces had a focal point to coordinate activities locally. Technical support from World Health Organization was provided throughout the process.

The health information system was updated to integrate new data collection forms. All health professionals were trained to collect data using these forms specifically designed for the Epilepsy Program. Data from health facilities were the main source of information for this study. It included information on patients’ consultations and socio-demographic characteristics, community interventions and availability of medication. Treatment gap estimates were calculated considering the new from 2015 to 2017 and the prevalence of epilepsy estimated by the only mental health prevalence survey conducted in the country in 2007.

## Results

**Fig. 1:**
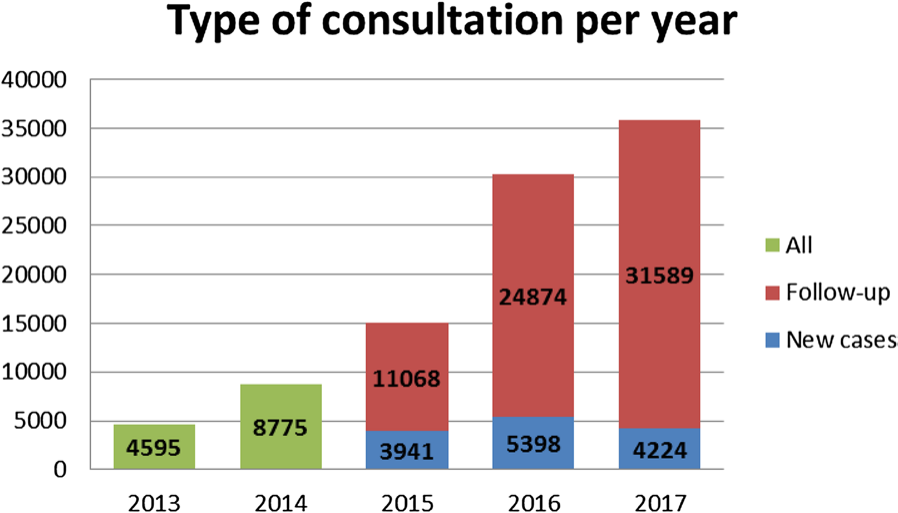
Number of epilepsy consultation per year

Prior to the Program’s inception in 2013, in all districts of the selected provinces a total of 4595 consultations were conducted with persons with epilepsy (PwE). In 2017 the 16 targeted districts alone conducted 35,813 consultations. From 2014 to 2017 a total of 89,869 consultations were conducted in these sites representing an increase of 67% (Fig. 1).

Since 2015, data on new cases started to be recorded. Between 2015 and 2017 a total of 13,563 new cases were seen in the selected primary health care facilities of which 54% were male. Around 53% of the new cases were children and adolescents under 18 years old.

The direct treatment gap estimates in the selected districts has reduced from 99% in 2015 to 96% in 2017. The cumulative new cases since 2015 were used for this estimates assuming that all new cases were already PwE without treatment as it was unavailable (Table 4).

**Table 4:** Treatment gap estimates

	2015	2016	2017
Population^a^	1,992,816	1,992,816	1,992,816
Epilepsy prevalence 1.6 (*n*1)	318,851	318,851	318,851
Nr. PwE (cumulative) (*n*2)	3941	9339	13,563
Treatment gap estimates (*n*1 − *n*2*/n*1 × 100)	99%	97%	96%

^a^Population of the health facilities catchment areas

On the basis of this evidence, in 2016 the Minister of Health signed an official directive to authorize the additional annual purchase of 1 ton of Phenobarbital 100 mg (2,954,960 pills) instead of the previous 10,000 tablets per year.

## Discussion

Training health professionals and having them changing their management of epilepsy was challenging. Implementing such changes in a health system can be cost-effective when evidence based strategies such as task-shifting and task-sharing are available and there is a commitment of all relevant stakeholders.

The mhGAP Program lead to changes in mental health care delivery but it requires changes in the general structure of the health system. In low-income settings, such as Mozambique, limited human resources can be redirected to effectively address mental disorders along with their main tasks.

By restructuring health care delivery using primary care providers with a task-shifting/sharing strategy, coverage of epilepsy patients increased with at large number of children and adolescents representing 53% of the new cases. A study in five African low- and middle-income countries (Ghana, Kenya, South Africa, Uganda and Tanzania) found that 51% of the people living with epilepsy evaluated were children and 69% of seizures began in childhood [13].

The epilepsy treatment gap in Mozambique is likely to be higher than in most low- and middle- income countries. About 80–98% of patients in the developing countries are untreated [10]. In Pakistan and Ethiopia it is as high as 98% [8]. In our study, we estimated a treatment gap of 99% meaning that at the beginning of the epilepsy Program only 1% of epileptic patients were receiving treatment. Implementing the mhGAP Epilepsy program led to a treatment gap of 96%, reducing the gap in these districts to a lower level than the average in similar populations. The reduction of the treatment gap, the increase in the number of patients and follow-up consultations constitute evidence that allowed the Ministry of Health to start scaling-up the Program for the entire country.

Ghana, Vietnam and Myanmar have implemented mhGAP with good results [13]. Mozambique has been innovative and integrated implementation in the primary health care services as part of the health system.

Involving community health workers in dissemination, cases identification and follow-up has proven to be cost-effective and well accepted by the target population. Zimbabwe has used this approach with the Friendship Bench, while Zambia, Rwanda and South Africa have also had various effective experiences with community health worker involvement. In Mozambique, interventions for reducing stigma and increase patients’ adherence to treatment were some of the most relevant outcomes of the community health workers’ involvement [13].

## Conclusion

Treatment gap for epilepsy in Mozambique is as high as 99%. Training non specialists and community health workers to deliver treatment and care for epilepsy increased by more than 67% the number of consultations in 3 years of effective implementation. The number of new cases increased more than twofold in the third year. The treatment gap reduction from 99 to 96% in 3 years is a remarkable achievement that needs to be replicated in the rest of the country. These results are encouraging for the Mental Health Department to continue using the task-shifting/sharing approach to reduce the treatment gap.

This Program revealed that more than half of patients are adolescents and children. This information is essential to advocate for changes to the type and presentation of anti-epileptic medicines purchased in order to include phenobarbital 15 mg and syrup. It also indicates a need for increasing awareness activities in schools and ante-natal consultation.

The increase of the quantity of anti-epileptic medicines procured by the Ministry of Health was a decision taken based on the preliminary results of the implementation of this Program.

Involving relevant stakeholders, continuous supervision and advocacy were also crucial to acceptance and sustainability of the Program.

The pilot implementation of mhGAP epilepsy in Mozambique generated important information for decision makers on alternative pathways for delivering care in community and primary health care settings using a task-shifting/sharing strategy.

## Data Availability

The datasets used and analyzed during the current study are available from the corresponding author on reasonable request.
